# Microstructure, transport and galvanomagnetic properties of Ni_48_Fe_12_Cr_40_ thin films

**DOI:** 10.3762/bjnano.17.87

**Published:** 2026-09-18

**Authors:** Roman Zavornitsyn, Mikhail Milyaev, Larisa Naumova, Irina Maksimova, Anastasia Pavlova, Tatyana Chernyshova, Vyacheslav Proglyado, Vladimir Ustinov

**Affiliations:** 1 Laboratory of Quantum Nanospintronics, M.N. Mikheev lnstitute of Metal Physics of Ural Branch of Russian Academy of Sciences, S. Kovalevskoi str., 18, Ekaterinburg 620108, Russia; 2 Department of Magnetism and Magnetic Nanomaterials, The Institute of Natural Sciences and Mathematics, Ural Federal University named after the first President of Russia B.N.Yeltsin, Kuybysheva str., 48, Ekaterinburg 620062, Russia

**Keywords:** Hall effect, magnetoresistance, NiFeCr alloy, permalloy, thin films

## Abstract

The microstructure, transport, and galvanometric properties of Ni_48_Fe_12_Cr_40_ thin films of various thickness, obtained by magnetron sputtering, were studied in the temperature range of 93–293 K. The Ni_48_Fe_12_Cr_40_ film with thickness of 5 nm has been found to be X-ray amorphous and exhibits a negative temperature coefficient of resistance. The observation of anisotropic magnetoresistance, as well as anomalous and planar Hall effect, indicates the presence of ferromagnetic ordering in this sample. The increase of the Ni_48_Fe_12_Cr_40_ film thickness to 8 nm leads to the crystallization and texture formation, which promotes more uniform distribution of Cr atoms in Ni–Fe matrix and suppression of the long-range ferromagnetic order.

## Introduction

NiFe-based alloys (permalloy) are a key material for spintronics and magnetic sensorics [1–2], due to their unique combination of properties, namely, high magnetic permeability, low coercive force, and significant anisotropic magnetoresistance (AMR). However, to solve new technological problems, such as reduction of the power consumption of memory elements [3], it is necessary to modify both transport and magnetic properties of the material. One of the most effective approaches to solving this problem is alloying NiFe binary alloys with transition metals, in particular chromium [3–4].

The introduction of chromium into the permalloy lattice leads to significant changes in the electronic structure and microstructure of the alloy, the nature of which depends on the concentration of alloying additive and film thickness [4–5]. According to the phase diagram for NiFeCr alloys [6], a bulk sample of Ni_48_Fe_12_Cr_40_ composition is characterized by the combined presence of α- and γ-phases, which have body-centered cubic (bcc) and face-centered cubic (fcc) structures, respectively. The preferential formation of α- or γ-phase in films and nanolayers of Ni_48_Fe_12_Cr_40_ alloy depends on the film thickness as well as on the substrate material or on the preceding layer of nanostructure. In [5], the bcc structure was discovered in the 5 nm thick film. We previously investigated the microstructure of Ni_48_Fe_12_Cr_40_ layer in superlattices exhibiting the giant magnetoresistance effect [7]. It has been shown that the bcc structure is formed in a 6.5 nm thick layer when sputtered on oxidized single-crystal silicon; however, when sputtered on a Ta sublayer with the same thickness of the Ni_48_Fe_12_Cr_40_ layer, the fcc structure is formed.

The substitution of nickel and iron atoms with chromium atoms, which have a larger atomic radius, causes a distortion of the crystal lattice, manifested in the increase of its cell parameter and the growth of microstresses [8]. The increasing structural disorder leads to the sharp increase in specific resistance and to a change of sign of the temperature coefficient of resistance (TCR) from positive to negative, which is characteristic of highly disordered systems [9–11].

Changes in the electron density of states and the microstructure after chromium alloying lead to the suppression of ferromagnetic ordering. Chromium atoms in the permalloy lattice have the magnetic moment oriented antiparallel to the magnetic moments of Ni and Fe; this leads to a compensation of total magnetic moment and the decrease of saturation magnetization [3]. It has been shown in [4], that at Cr impurity concentration of 10%, the saturation magnetization of permalloy decreases by 30%. The increase of Cr concentration leads to the monotonous decrease of the Curie temperature (*T*_c_). For a (Ni_80_Fe_20_)_100−_*_x_*Cr*_x_*(11nm) film obtained by magnetron sputtering, *T*_c_ varies within 800–250 K in the concentration range 0 ≤ *x* ≤ 35% [4]. In [12], it is reported that at high concentrations of Cr (>20–30%), the system can undergo phase transitions to spin glass or paramagnetic state at room temperature, exhibiting complex magnetic behavior.

The use of galvanomagnetic effects, the Hall effect in particular, as a sensitive tool for diagnosing the magnetic phase transformations is of particular interest. Since the anomalous Hall component is directly proportional to the magnetization, the presence or absence of anomalous contributions makes it possible to track the evolution of magnetic state of the system under conditions where classical magnetometry data can be ambiguous [12]. In addition, alloying Ni–Fe systems with chromium leads to the change of electron band structure and contribution of various groups of charge carriers. Experimentally it is demonstrated by the change of Hall constant sign. In NiFeCr alloys at the increase of Cr concentration from 12 to 18%, the transition from negative values of ordinary (*R*_0_) and anomalous (*R*_S_) Hall coefficients, characteristic of pure Ni or NiFe, to positive ones was observed [2,13].

The effect of low concentrations of Cr alloying additives on the electrical and magnetic properties of NiFe has been studied in sufficient details. However, the behavior of heavily alloyed alloys remains a subject of debate. For Ni–Fe–Cr system, the critical Cr concentration suppressing the long-range magnetic order is approximately 35–45% [4]. At such concentrations of the alloying element, the alloy presents a highly disordered system. The study of transport and magnetic properties of ultrathin layers (<10 nm) of heavily alloyed NiFeCr alloys, which are the functional materials for modern spintronic devices, is of special interest. In particular, the use of Ni_48_Fe_12_Cr_40_ alloy as a buffer layer [5,7,14–15] in the synthesis of magnetic nanostructures leads to the increase of magnetoresistance and decrease of hysteresis due to the formation of pronounced (111) crystal texture and the increase of grain size.

In this paper, the correlation between the microstructure and galvanomagnetic properties of Ni_48_Fe_12_Cr_40_ films has been investigated. Basing on the analysis of magnetoresistance measurement results, anomalous and planar Hall effect, a conclusion about the magnetic ordering of Ni_48_Fe_12_Cr_40_ thin films has been made.

## Results and Discussion

### Microstructure and morphology of Ni_48_Fe_12_Cr_40_ films surface

**Figure 1:**
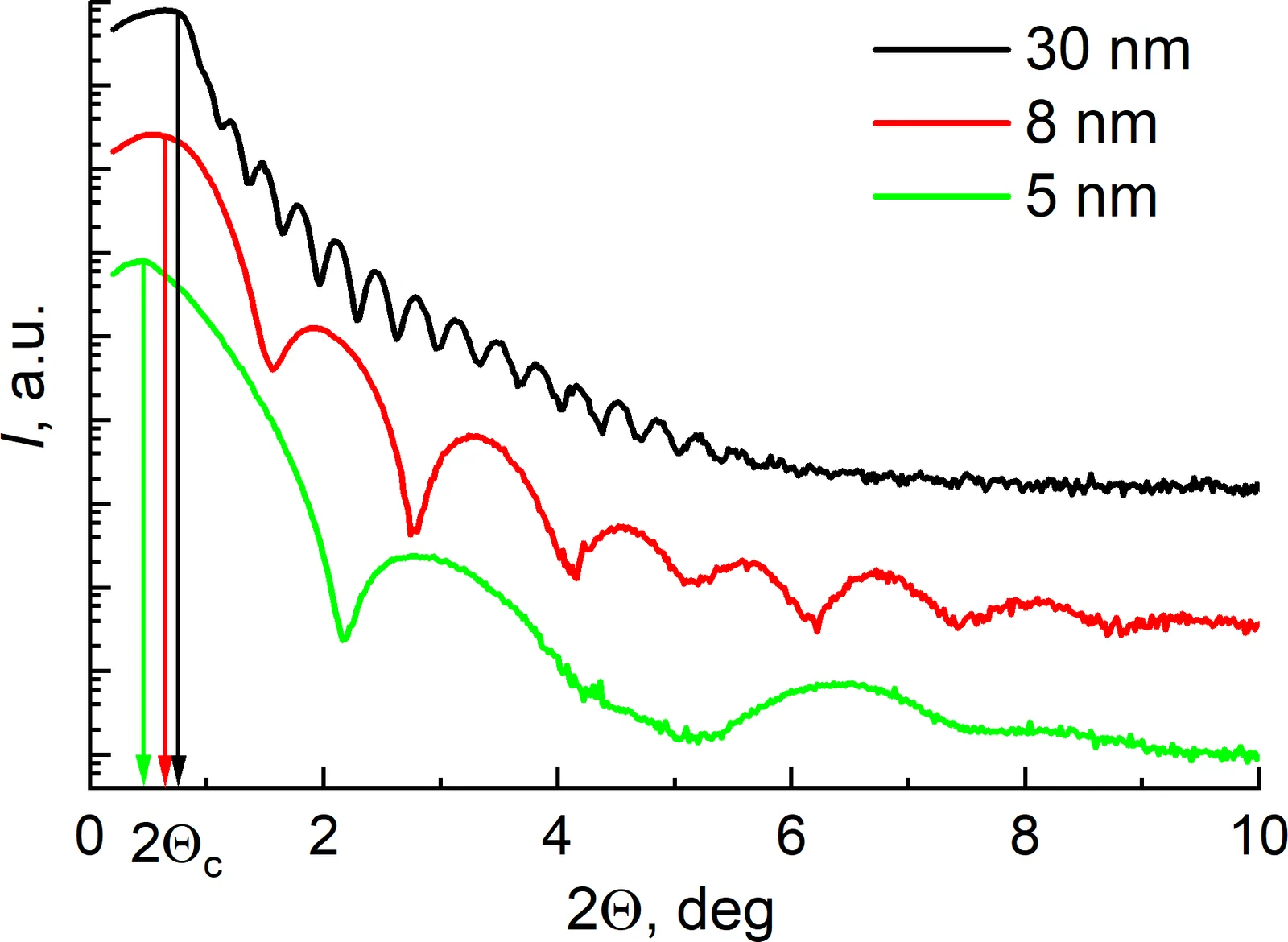
Results of X-ray reflectometry of NiFeCr films with thickness of 5, 8, and 30 nm.

Figure 1 shows the results of X-ray reflectometry for films of different thickness. The thickness was estimated from the oscillation period of the reflectometry curves obtained for the films *t*_NiFeCr_ = 8 and 30 nm. The obtained values *t*_NiFeCr_ = 7.9 and 30.5 nm are close to the nominal thickness specified during sputtering. For the films of different thicknesses, approximately equal decrease in the intensity of reflected radiation is observed with the increase of angle 2Θ. Consequently, the films have similar values of surface roughness.

The important characteristic that can be obtained from the results of X-ray reflectometry is the critical angle (Θ_c_) of total external reflection of X-rays from the surface of the analyzed film [16–17]. If the angle between the incident radiation and the sample surface Θ < Θ_c_, the radiation is completely reflected. At Θ > Θ_c_ the radiation is refracted and enters the sample, and a decrease in the intensity of reflected signal with the increase of angle 2Θ is detected (Figure 1). For the films with thicknesses of 8 and 30 nm, the Θ_c_ values coincide practically, and for the film with 5 nm thickness the critical angle is noticeably smaller. This difference may be due to the fact that electron density and, accordingly, the density of the film material at *t*_NiFeCr_ = 5 nm is less than at *t*_NiFeCr_ = 8 and 30 nm.

**Figure 2:**
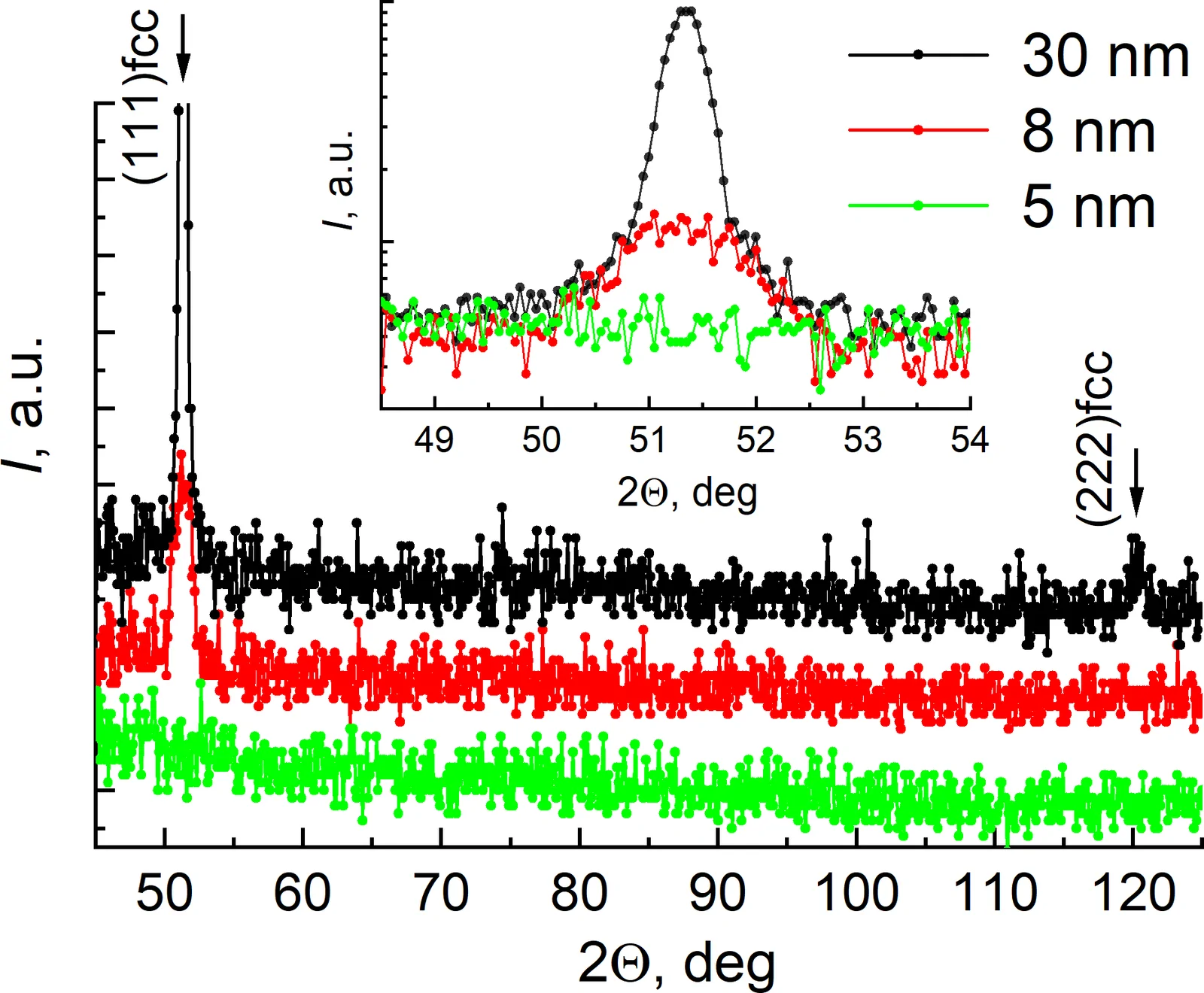
X-ray diffraction results for NiFeCr films with 5, 8, and 30 nm thickness. The inset shows the (111) fcc reflection, obtained by measuring in a smaller angular range and at longer exposure time.

Figure 2 shows the X-ray diffraction patterns obtained for the films of different thicknesses. For *t*_NiFeCr_ = 5 nm, no diffraction peaks were detected in the entire studied angular range. Basing on the X-ray amorphism of this sample, it can be assumed that the 5 nm thick film has a disordered fine-crystalline microstructure. With the increase of the film thickness, the (111) and (222) reflections, corresponding to the fcc lattice, appear in the diffraction pattern. Texture investigations have shown that the widths at half-height of the rocking curve (ω-scan) around the (111) peak are 9° and 11° for films with thicknesses of 8 and 30 nm, respectively. Therefore, for *t*_NiFeCr_ = 8 and 30 nm the axial texture ⟨111⟩ is formed, and the average angles of texture axis deviation from the normal to the film surface are 9° and 11°.

**Figure 3:**
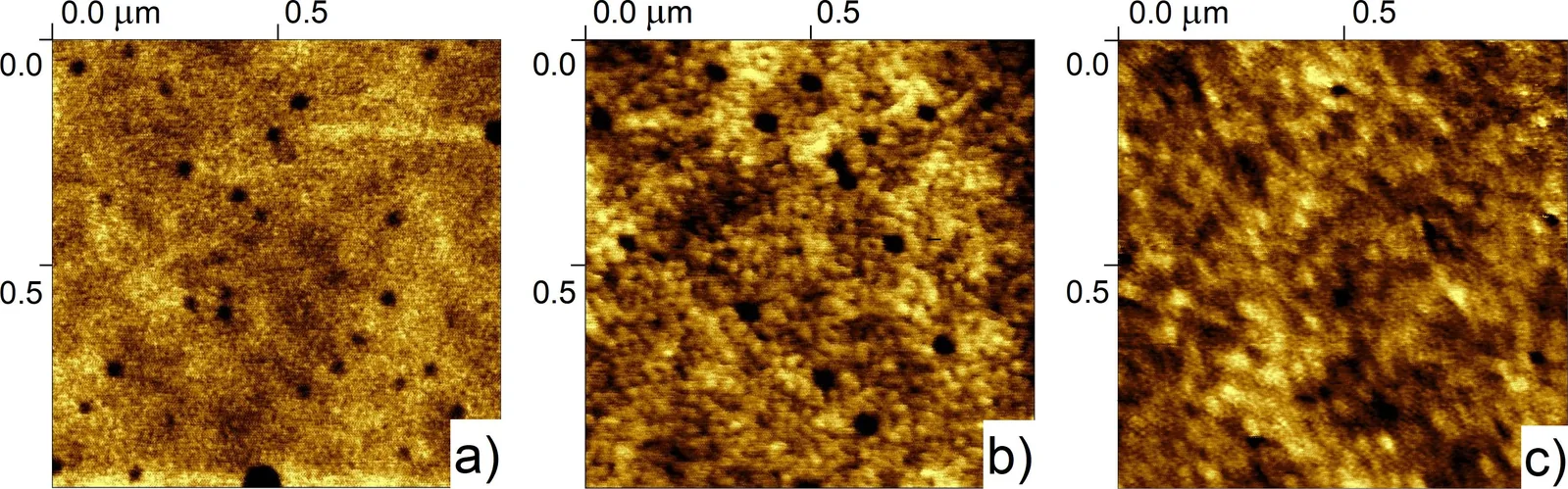
AFM images of the surface of films with thickness of (a) 5 nm, (b) 8 nm, and (c) 30 nm.

The images obtained using atomic force microscopy (AFM, Figure 3) show that the surface of 5 nm thick films looks different from the surface of thicker films *t*_NiFeCr_ = 8 and 30 nm. The difference is in the finer dispersion and a larger number of pores. The observed surface morphology comes to agreement with the conclusions, made according to the X-ray investigation results, about the X-ray amorphous disordered microstructure and lower density of the material.

Crystal grains with the average lateral size of approximately 30 nm are seen on the surface of the *t*_NiFeCr_ = 8 nm sample, which has a polycrystalline fcc structure and the most perfect ⟨111⟩ texture. With the increase of film thickness to *t*_NiFeCr_ = 30 nm, the size of crystallites increases. The surface roughnesses, estimated as the root-mean-square deviation (rms) and measured on an area of 1 µm × 1 µm, are rms = 0.24, 0.22, and 0.20 nm. The obtained rms values are very close to each other, which is in agreement with the results of X-ray reflectometry.

The surface roughness of the glass substrates is rms = 0.2 nm. The surface roughness of sputtered films usually increases with film thickness. In our X-ray reflectometry and AFM measurements the films show similar roughness values regardless of thickness increase. Probably, in this case, the contributions of two opposite trends that occur with an increase in the thickness of the film are added. Namely, reduction in the number of pores, which leads to roughness smoothing and grain growth the polycrystalline films, which often increases surface roughness.

Thus, microstructure and morphology of the film surface at *t*_NiFeCr_ = 5 nm differ significantly from those obtained for *t*_NiFeCr_ = 8 and 30 nm. In particular, at *t*_NiFeCr_ = 8 and 30 nm, the polycrystalline films have fcc structure and axial texture ⟨111⟩; the X-ray-amorphous *t*_NiFeCr_ = 5 nm film has a disordered microstructure, small grain size, larger number of pores and lower material density and electron density than the *t*_NiFeCr_ = 8 and 30 nm samples.

### Specific electrical resistance and temperature coefficient of resistance for Ni_48_Fe_12_Cr_40_ films

**Figure 4:**
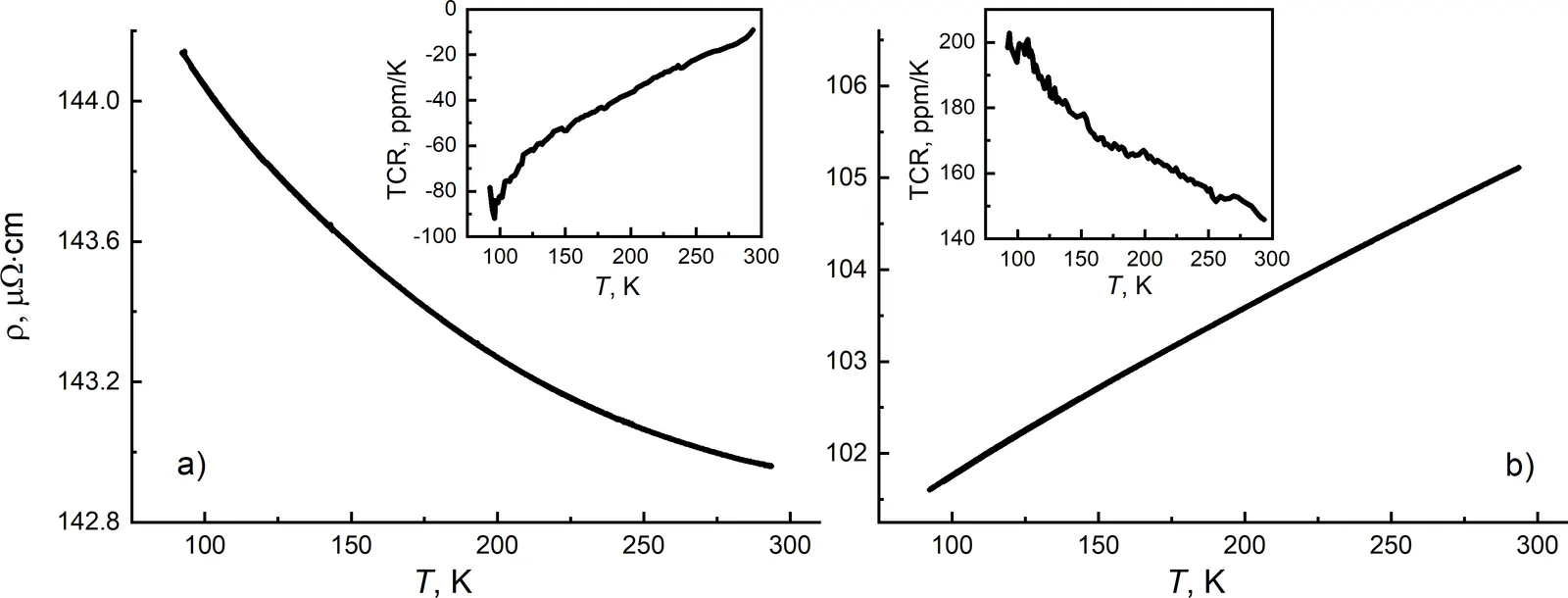
Temperature dependences of specific electrical resistance and temperature coefficient of resistance for Ni_48_Fe_12_Cr_40_ films with thicknesses of (a) 5 nm and (b) 8 nm.

Figure 4 shows the temperature dependences of electrical resistance and TCR of Ni_48_Fe_12_Cr_40_ films with thicknesses of 5 and 8 nm. The specific resistance of the studied Ni_48_Fe_12_Cr_40_ films exceeds significantly the values common to polycrystalline Ni_48_Fe_12_Cr_40_ films (20–30 µΩ·cm). The growth in electrical resistance, due to the heavy alloying with Cr, leads to a significant increase of impurity electron scattering. An additional contribution comes from the size effect, associated with the scattering of charge carriers on the thin film surfaces.

For the Ni_48_Fe_12_Cr_40_(5nm) film, a negative TCR was observed, the decrease of temperature was accompanied by an increase in specific electrical resistance. This situation is typical for the highly disordered systems and is caused by the strong scattering of charge carriers due to the small free path length [11]. With the increase in Ni_48_Fe_12_Cr_40_ film thickness from 5 to 8 nm, the change in TCR sign from negative to positive was observed, and the temperature dependence of the specific electrical resistance of the Ni_48_Fe_12_Cr_40_(8nm) film took the typical form of metals, that is, a decrease in temperature was accompanied by a decrease in the value of specific electrical resistance. The increase in Ni_48_Fe_12_Cr_40_ film thickness also led to the increase in the absolute value of TCR, caused by the increase of grain size [18]. The increase in the absolute value of TCR observed at the decrease in temperature for Ni_48_Fe_12_Cr_40_ films of 5 and 8 nm thickness is apparently associated with the increase in the free path length of charge carriers due to the decrease of phonon scattering [19–20].

### Magnetoresistance of Ni_48_Fe_12_Cr_40_ thin films

**Figure 5:**
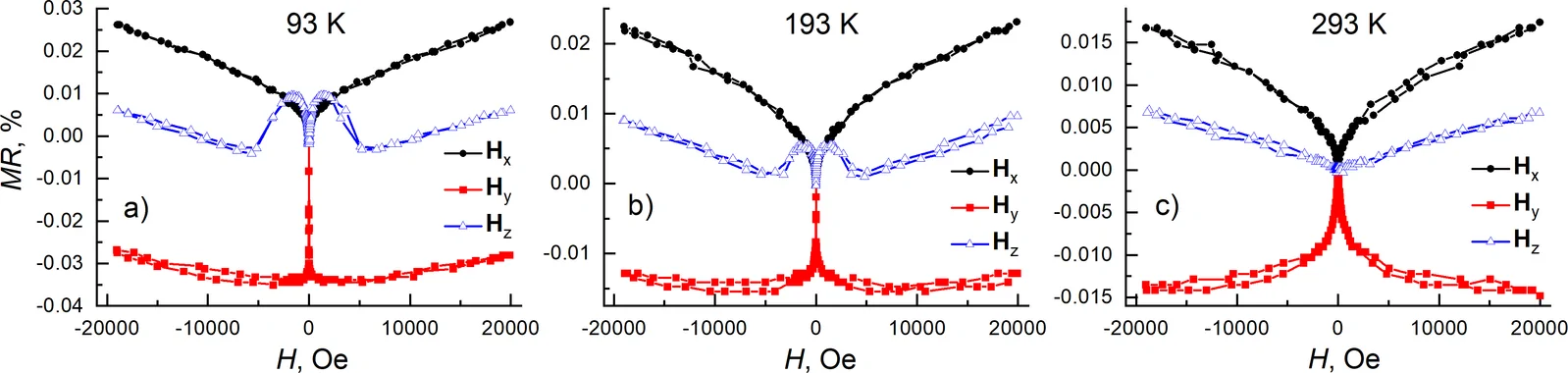
Field dependences of magnetoresistance for Ni_48_Fe_12_Cr_40_(5nm) at different orientations of external magnetic field at temperatures of (a) 93 K, (b) 193 K, and (c) 293 K.

Figure 5 shows the field dependences of magnetoresistance for the Ni_48_Fe_12_Cr_40_(5nm) film, measured at different temperatures and external magnetic field orientations. A possible explanation of the observed magnetoresistance is the existence of ferromagnetic ordering in the Ni_48_Fe_12_Cr_40_(5nm) film. Anisotropic magnetoresistance is maximal in case of collinear orientation of the ferromagnet magnetic moment and electric current flowing through the sample, and minimum at orthogonal orientation. It is reported in [4], that NiFeCr alloys can have ferromagnetic ordering, and the Curie temperature depends on the Cr concentration introduced into NiFe composition and on the film thickness.

Let us analyze the nature of the field dependences of magnetoresistance for the Ni_48_Fe_12_Cr_40_(5nm) film at different orientations of external magnetic field.

#### Longitudinal magnetoresistance MR*_x_* in an H*_x_* field

Longitudinal magnetoresistance is positive in the temperature range of 93–293 K and is caused by the external magnetic field that aligns the magnetic moments in the film along the electric current. With the increase of temperature, the value of MR*_x_* decreases from 0.026% to 0.016% due to the weakening of magnetic ordering (Figure 5).

#### Transverse magnetoresistance MR*_y_* in an H*_y_* field

In the dependence MR*_y_*(*H*) two sections can be seen, namely, (1) in the range of low fields a sharp drop of magnetoresistance occurs, and (2) with further increase in the magnetic field, a monotonic change of magnetoresistance is observed and the slope of *MR*_y_(*H*) dependence depends on the temperature (Figure 5). In an **H***_y_* field, magnetic moments are ordered orthogonally to the electric current. This causes a sharp decrease of magnetoresistance MR*_y_* at low fields. The value of magnetoresistance decreases with the increase of temperature, which is typical for AMR, since the magnetic moment decreases and the contribution of phonon scattering increases. It is interesting to see that at *T* = 293 K, the MR*_y_* value continues to decrease with the increase of magnetic field. This behavior may be attributed to an increased contribution of magnon magnetoresistance (MMR) [21–22]. As the temperature rises, the concentration of thermally excited magnons increases, leading to an enhanced contribution of electron–magnon scattering to the electrical resistance. The suppression of these thermal magnons by an external magnetic field results in a more pronounced decrease in resistance. Although MMR is isotropic, it manifests most distinctly in the transverse (**H***_y_*) magnetic field orientation. In the longitudinal (**H***_x_*) and out-of-plane (**H***_z_*) orientations, the MMR is masked by the more dominant anisotropic magnetoresistance and ordinary magnetoresistance (OMR) caused by the Lorentz force, respectively.

Conversely, as the temperature decreases, the magnetoresistance begins to increase in the high-field region. This transition is due to a significant reduction in the MMR contribution resulting from a lower concentration of thermal magnons. Consequently, the positive OMR contribution becomes dominant, leading to the observed increase in resistance [23].

#### Orthogonal magnetoresistance MR*_z_* in an H*_z_* field

In the field range of ±5 kOe there are features, on the field dependences *MR*_z_ at *T* = 93 K, that become less noticeable with the temperature increase and disappear at *T* = 293 K (Figure 5). The further increase in the magnetic field is accompanied by the increase of magnetoresistance value. The observed features of MR*_z_*(*H*) in the field range of ±5 kOe at *T* = 93 K can occur due to the exit of magnetic moment from the film plane. In fields up to ±1.5 kOe, a growth of the MR*_z_* value is observed. A possible reason is the non-strictly orthogonal orientation of the **H**_z_ field relative to the film plane. The appearance of non-zero field projection in the film plane can lead to the ordering of magnetic moments collinearly to the electric current, which is accompanied by an increase of magnetoresistance (AMR). At further increase of the magnetic field (up to ±5 kOe), the magnetoresistance value decreases, which is caused by the exit of magnetic moments from the film plane and the formation of the orthogonal configuration of magnetization and electric current. The saturation field (≈5 kOe in this case) corresponds to the demagnetizing field *H*_sat_ = 4π*M*_S_. The further increase of the magnetic field is accompanied by the linear growth of MR*_z_*, which is due to the OMR contribution.

With the temperature increase to 293 K, the peculiar features of magnetoresistance at low fields disappear, which can be the consequence of a decrease in saturation magnetization due to the vicinity of the alloy to the ferromagnetic–paramagnetic phase transition. According to [24], the segregation of chromium atoms at the boundaries of nanoparticles has been observed in NiCr alloys. In thin films, it can lead to the formation of ferromagnetic clusters with low Cr content, separated by Cr-rich boundaries. It is probable that in the 5 nm thick film under investigation, the observed AMR (and the presence of long-range ferromagnetic order) is also due to the chemical inhomogeneity and clustering of Ni and Fe atoms.

**Figure 6:**
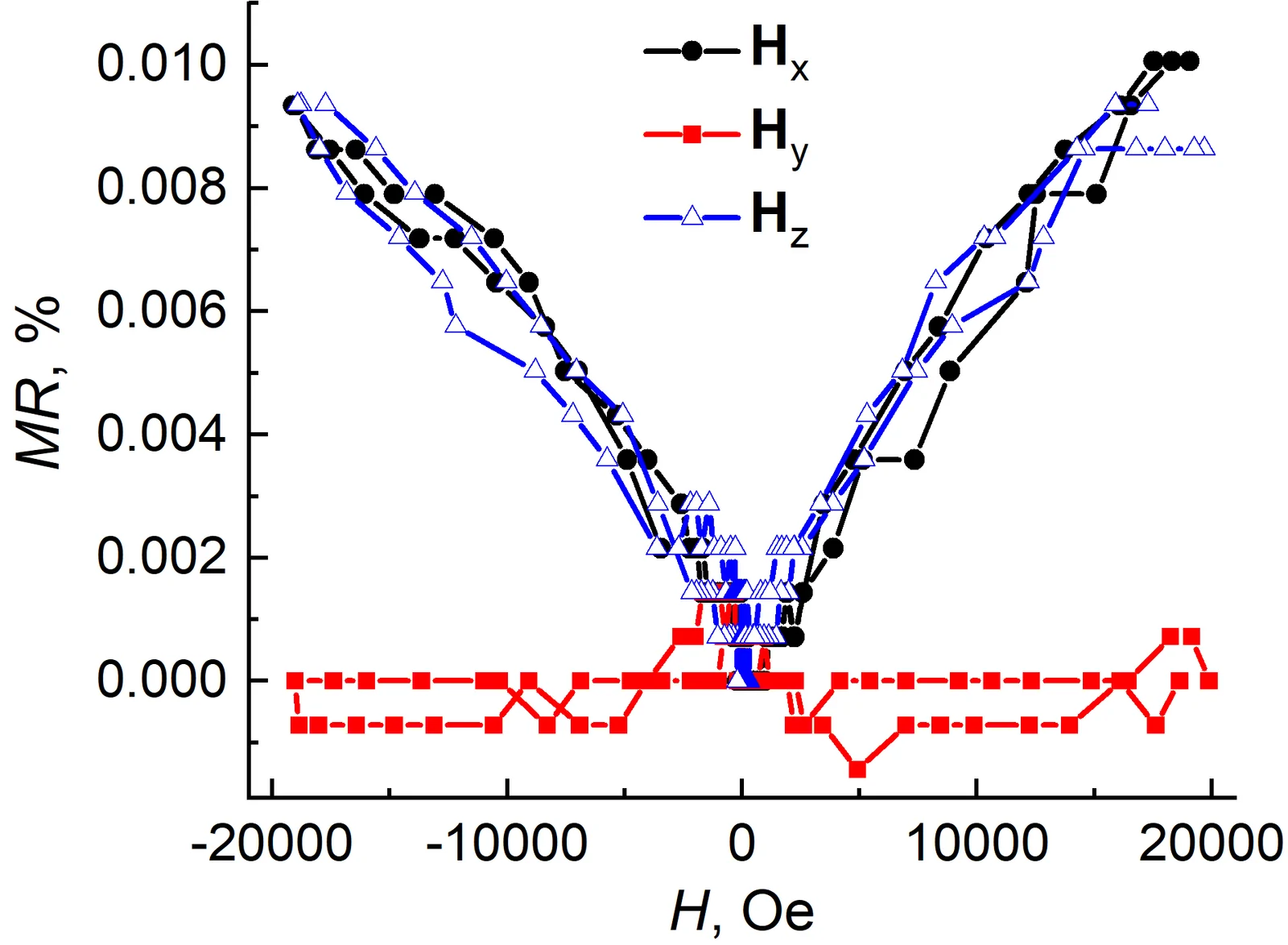
Field dependences of magnetoresistance for Ni_48_Fe_12_Cr_40_(8nm) film at different orientations of the external magnetic field at 93 K.

With the increase of Ni_48_Fe_12_Cr_40_ film thickness from 5 to 8 nm, a change in the shape of the field dependence of magnetoresistance and a decrease of the effect value are observed (Figure 6). The possible reason is the evolution of the film microstructure.

A peculiar feature of the field dependences of magnetoresistance of the Ni_48_Fe_12_Cr_40_(8nm) film is an almost complete coincidence of the curves for longitudinal (**H***_x_*) and orthogonal (**H***_z_*) orientations of the magnetic field; MR*_x_*(*H*) and MR*_z_*(*H*) demonstrate a linear positive growth with increasing *H*. As shown in [25], the positive, linear in the field and isotropic magnetoresistance is a characteristic feature of thin films of granular ferromagnetic metals (Ni, Fe, Co). The dominant mechanism in such systems is the ordinary magnetoresistance caused by the Lorentz force and the effects of path curvature of the current flow under conditions of an inhomogeneous medium [26].

The transverse magnetoresistance of the film (in an **H***_y_* field) does not depend practically on the magnetic field strength. The reason may be in the mutual compensation of two contributions, that is, negative AMR and positive OMR. However, unlike the orthogonal geometry (**H***_z_*), the OMR contribution is significantly weakened by the size effect and charge carriers scattering on the thin film surfaces. With the temperature increase to 293 K the nature of field dependences of magnetoresistance is preserved, and the magnitude of effect decreases by ≈1.5 times, which is associated with the increase of phonon scattering.

Unlike the 5 nm thick film, the Ni_48_Fe_12_Cr_40_(8nm) film does not exhibit a pronounced AMR. According to the microstructure investigation data, the increase of film thickness is accompanied by crystallization and texture formation. This process, probably, contributes to the more uniform distribution of Cr atoms in the NiFe matrix and to the effective suppression of magnetic moments.

It is important to note that the geometry where the magnetoresistance for the Ni_48_Fe_12_Cr_40_ film of 8 nm thickness is observed coincides with the configuration required for the observation of Hanle magnetoresistance [27]. Regarding this, the suppression of spin accumulation can be considered as an alternative interpretation of the observed phenomena. To determine the dominant mechanism for certain, additional studies are necessary.

### Hall effects in Ni_48_Fe_12_Cr_40_ thin films

**Figure 7:**
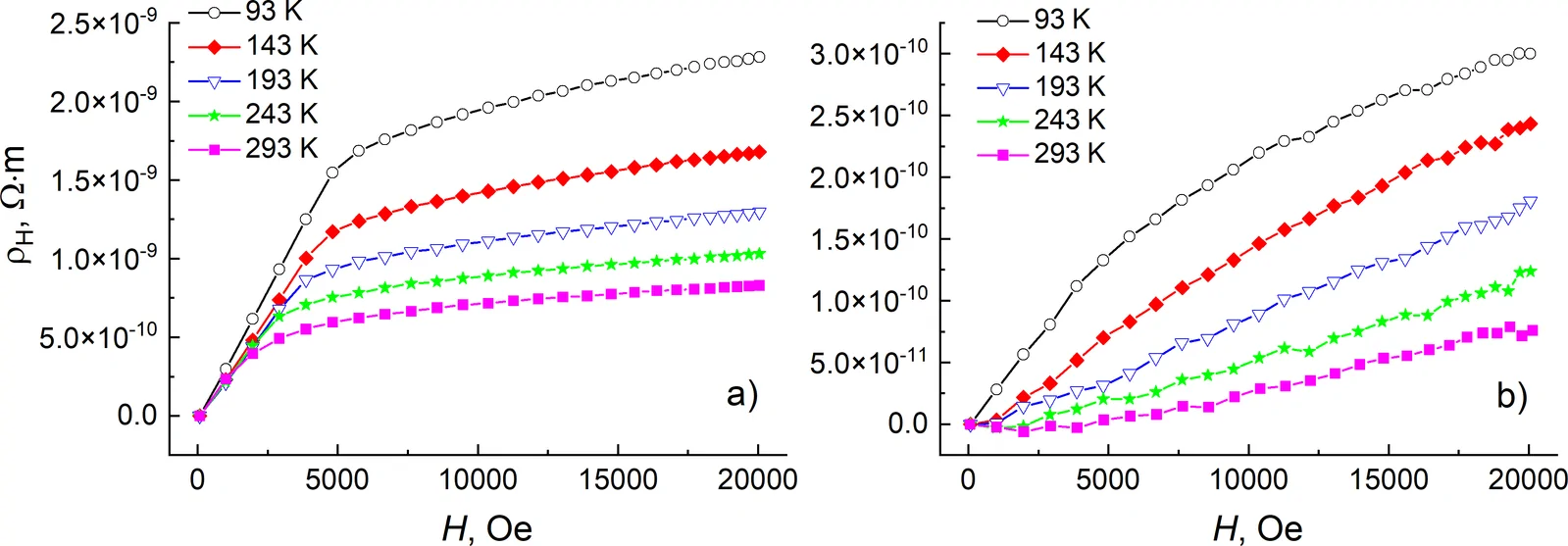
Dependences ρ_H_(*H*) for Ni_48_Fe_12_Cr_40_ films with thicknesses of (a) 5 nm and (b) 8 nm.

Figure 7 shows the field dependences of the Hall resistance for Ni_48_Fe_12_Cr_40_ films with thicknesses of 5 and 8 nm. The dependences ρ_H_(*H*) for Ni_48_Fe_12_Cr_40_(5nm) film demonstrate the behavior characteristic of ferromagnetic materials with dominant contribution from the anomalous Hall effect [28–30]. In the region of low fields (<5 kOe for *T* = 93 K) a linear increase in the signal is observed, associated with the process of aligning the magnetic moments orthogonally to the film plane. Upon reaching saturation (≈5 kOe, *T* = 93 K) a bend is observed in the dependence, followed by a more graduate increase of ρ_H_ with increasing magnetic field. It is important to note that the saturation field determined from the bend point of ρ_H_(*H*) at *T* = 93 K coincides quantitatively with the saturation field of the magnetoresistance in the orthogonal magnetic field geometry (**H***_z_*) at *T* = 93 K (Figure 5a). This correlation confirms that both galvanomagnetic effects (anisotropic magnetoresistance and anomalous Hall effect) are associated with the exit of magnetic moments from the plane at the increase of *H*_z_.

Analysis of the ρ_H_(*H*) dependences shows that the bend point corresponding to the saturation field shifts to the lower field area at the increase of temperature. The magnitude of this field makes it possible to estimate the saturation magnetization of the material (*H*_sat_ = 4π*M*_S_). As a result, for the Ni_48_Fe_12_Cr_40_(5nm) film, the value of *M*_S_ decreases from 430 to 200 emu/cm^3^ with the growth of temperature from 93 to 293 K. For comparison, the saturation magnetization of Ni_80_Fe_20_ permalloy films is of the order of 800 emu/cm^3^ and is practically constant in the specified temperature range due to the distance from the Curie temperature (≈850 K) [31–33]. The significant decrease in magnetization in Ni_48_Fe_12_Cr_40_(5nm) is due to the dilution of the magnetic subsystem with chromium atoms. Moreover, the pronounced temperature dependence of *M*_S_ indicates the sharp decrease in Curie temperature of the alloy compared to permalloy and is a sign of the system’s closeness to the magnetic phase transition.

For the Ni_48_Fe_12_Cr_40_(8nm) film, the character of dependences approaches the linear form, which is typical of nonmagnetic metals (Figure 7b). At low temperatures (93–143 K), a weak nonlinearity of the signal is still visible in the curves. The residual contribution from the anomalous Hall effect indicates the preservation of ferromagnetic ordering in the film in this temperature range; however, the amplitude of this contribution is smaller by an order of magnitude than in the Ni_48_Fe_12_Cr_40_(5nm) film. With a further increase in temperature (193–293 K), the field dependences of the Hall resistance become linear.

The decrease of the slope of ρ_H_(*H*) dependences with increasing temperature is an important peculiarity. As a rule, for nonmagnetic metals, the slope of ρ_H_(*H*) (and Hall coefficient, respectively) does not depend on temperature [34]. In this case, the observed temperature dependence of the slope indicates that the measured signal has a contribution related to magnetism. Apparently, despite a more uniform distribution of Cr atoms in the matrix compared to the Ni_48_Fe_12_Cr_40_(5nm) film, the magnetic ordering in the local clusters is preserved; however, their collective response to the external magnetic field weakens due to the growth of thermal fluctuations.

It should be noted separately that for the studied Ni_48_Fe_12_Cr_40_ films of 5 and 8 nm in thickness, the slope coefficients of ρ_H_(*H*) dependence (and hence the Hall constant *R*_0_) have a positive sign. This result is in agreement with the data for pure Cr [35–36] and disordered Ni-based alloys [2,34], which exhibit hole conductivity. Quantitative estimation of the Hall coefficients showed that for 5 and 8 nm films, the Hall constant *R*_0_ varies within the range of (1.2–3.6) × 10^−10^ m^3^/C and (5.5–8.4) × 10^−11^ m^3^/C, respectively, in the temperature range of 93–293 K. The coefficient of the anomalous Hall effect, *R*_S_, for the 5 nm film reaches values of (2.4–2.9) × 10^−9^ m^3^/C. The obtained experimental data are in good qualitative and quantitative agreement with the results of [13], where positive signs of the coefficients *R*_0_ = 4.2 × 10^−11^ m^3^/C and *R*_S_ = 2.3 × 10^−9^ m^3^/C were also observed for the bulk Ni_73.5_Fe_8_Cr_18.5_ alloy. The authors conclude that in this alloy, the dominant mechanism of the anomalous Hall effect is a lateral displacement (side jump), which prevails in the systems with strong scattering, namely, with high specific resistance and small length of free path. Taking into account the good correlation of the *R*_S_ estimation for Ni_48_Fe_12_Cr_40_(5nm) with the data of [13], it can be assumed that transport properties of the investigated films are determined by the same scattering mechanisms as in the bulk NiFeCr alloys.

**Figure 8:**
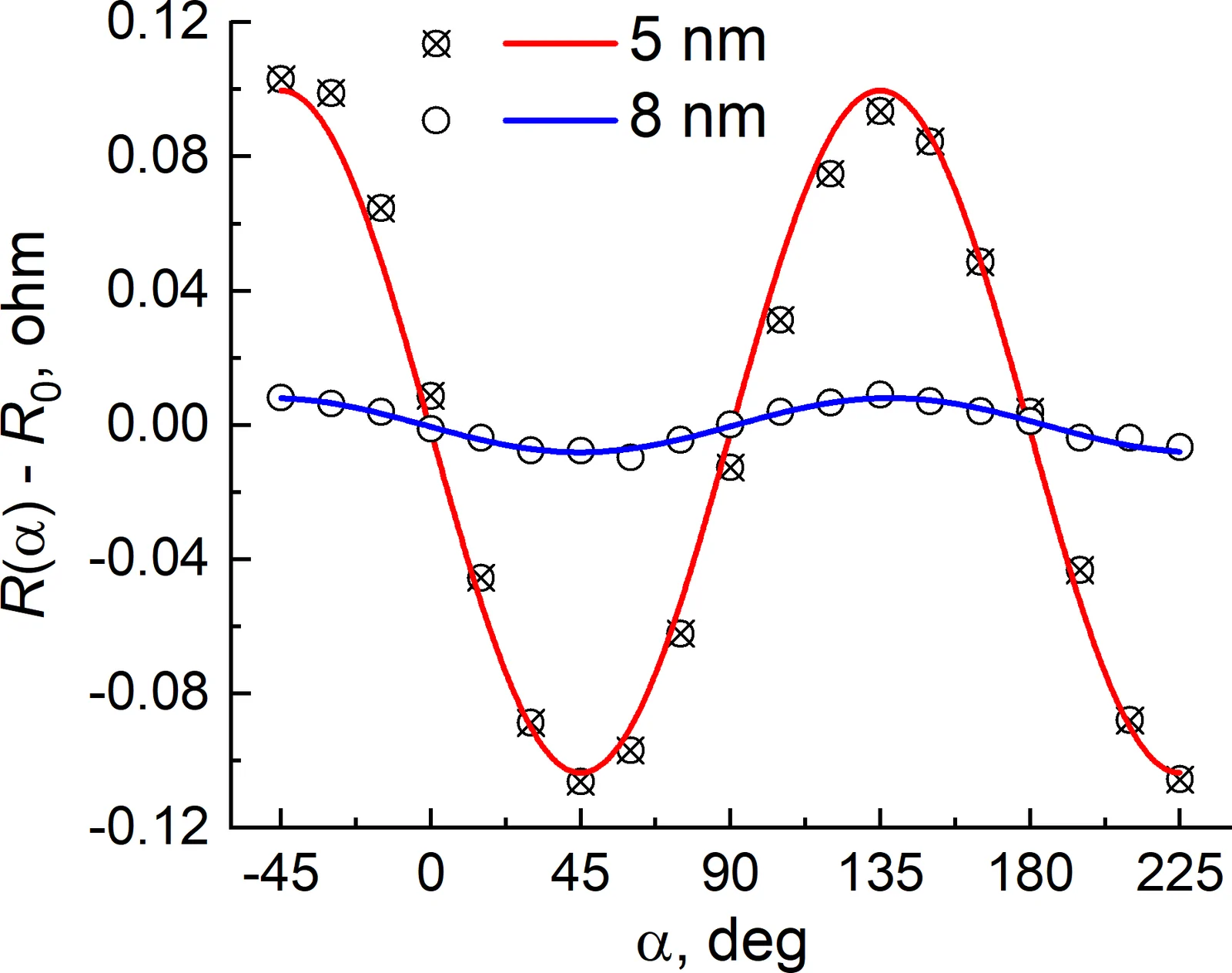
Angular dependence of planar Hall effect amplitude in the field *H* = 20 kOe for Ni_48_Fe_12_Cr_40_ films of 5 and 8 nm thickness at *T* = 93 K. α is the angle between the electric current direction and the external magnetic field; the symbols indicate the experimental data, the lines are approximations.

The results of measuring the planar Hall effect (PHE), which is connected with anisotropic magnetoresistance in ferromagnets, confirm the conclusions about the magnetic ordering of the investigated films. Figure 8 shows the angular dependences of PHE amplitude in an external field of *H* = 20 kOe, applied in the plane for 5 and 8 nm films at *T* = 93 K. The experimental data are successfully approximated by the periodic function sin(2α), and the extrema are observed at α = −45°, 135° (maximum) and α = 45°, 225° (minimum), which is typical of the planar Hall effect [37].

The Ni_48_Fe_12_Cr_40_(5nm) film exhibits a pronounced amplitude of PHE, which together with the presence of anomalous contribution to the Hall effect and the observation of anisotropic magnetoresistance, confirms the assumption of ferromagnetic ordering of the sample. The PHE amplitude for Ni_48_Fe_12_Cr_40_(8nm) is by one order of magnitude lower, which indicates the suppression of ferromagnetic ordering. Moreover, the presence of a weak but noticeable PHE signal, the nonlinearity of Hall curves at low temperatures, and the dependence of their slope on the temperature can be a mark of existence of local magnetic clusters.

## Conclusion

Complex studies of microstructure, as well as the transport and galvanomagnetic properties of Ni_48_Fe_12_Cr_40_ thin films synthesized by magnetron sputtering on glass substrates allow us to draw the following conclusions:

The 5 nm film thick is X-ray amorphous and has a disordered fine-crystalline microstructure. The 8 and 30 nm thick films are polycrystalline. As the film thickness increases, a face-centered cubic structure and ⟨111⟩ axial texture is formed, along with the increase of grain size and material density. All studied films have similar values of surface roughness.

The correlation between microstructure and transport properties is revealed. The negative temperature coefficient of resistance is observed for the amorphous film of 5 nm thickness due to the strong scattering of charge carriers in the disordered systems. The increase of film thickness leads to sign inversion of the temperature coefficient of resistance and increase in its absolute value, which is connected with the increase of grain size.

The observation of anisotropic magnetoresistance, anomalous contribution to the Hall effect, and the pronounced amplitude of the planar Hall effect for the 5 nm thick film allow us to make a conclusion that this sample has a ferromagnetic ordering.

The saturation magnetization of the 5 nm thick film decreases from 430 to 200 emu/cm^3^ with the temperature increasing from 93 to 293 K, which is two to four times lower than the characteristic values of pure permalloy. The sharp temperature dependence of saturation magnetization indicates a significant decrease of the Curie temperature of the alloy compared to permalloy and is the evidence of the system’s closeness to a magnetic phase transition.

The increase of the film thickness from 5 to 8 nm leads to a more uniform distribution of Cr atoms in the Ni–Fe matrix and suppression of the long-range ferromagnetic order. However, the peculiarities of field dependences of the Hall resistance and the presence of the planar Hall effect for the 8 nm thick film indicate the existence of local magnetic clusters.

## Experimental

Ni_48_Fe_12_Cr_40_ films with thickness of *t*_NiFeCr_ = 5, 8, and 30 nm were fabricated by magnetron sputtering (high vacuum precision magnetron setup MPS-4000-C6 (ULVAC Inc., Japan)). We used Corning glass 25 × 25 mm square substrates with average surface roughness of 0.2 nm. Sputtering was carried out at room temperature and 100 W power using a Ni_48_Fe_12_Cr_40_ alloy target. The target, purchased from Girmet Ltd (Russia), was prepared from high-purity source materials: Ni (99.99%), Cr (99.95%), and Fe (≥99.9%), providing an overall nominal purity of at least 99.9%. The exact stoichiometry of the target was confirmed by energy-dispersive X-ray spectroscopy analysis as Ni (48.05 atom %), Fe (12.17 atom %), and Cr (39.78 atom %). To minimize the stoichiometric shift in Ni_48_Fe_12_Cr_40_, the targets were presputtered for 40 s with a closed shutter prior to the deposition of each sample. This procedure ensures a steady-state sputtering regime and guarantees the chemical homogeneity and identical composition of the films, regardless of their thickness. The pressure of the working gas (argon, 99.999% purity) in the sputtering chamber was 0.1 Pa with a residual gas base pressure of 5 × 10^−7^ Pa.

**Figure 9:**
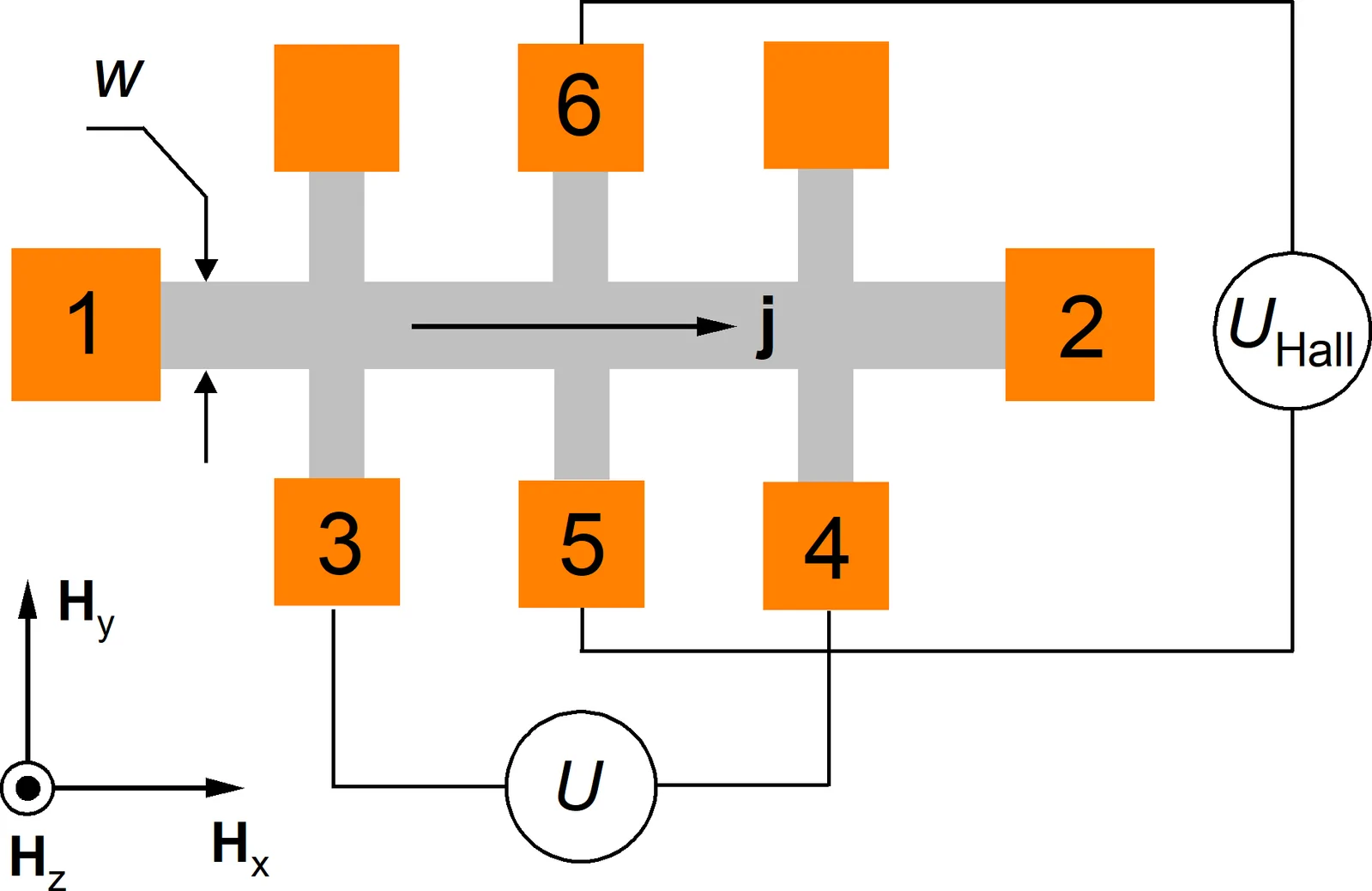
Schematic image of the Hall bridge.

To study the galvanomagnetic properties of Ni_48_Fe_12_Cr_40_ thin films, Hall bridge-shaped micro-objects (Figure 9) were patterned using a SUSS MicroTec MJB4 optical photolithography system (Germany). The current channel width was *w* = 200 µm, and the distance between current (1, 2) and potential (3, 4) contact pads were 2700 and 2200 µm, respectively. The electrical resistance of the micro-objects was measured in the temperature range of 93–293 K using a setup based on an electromagnet, a pumping cryostat and a temperature controller.

When measuring the magnetoresistance, the external magnetic field was applied collinearly (**H***_x_*) and orthogonally (**H***_y_*) to the direction of the electric current in the film plane, as well as orthogonally to the direction of the electric current and the film plane (**H***_z_*). Magnetoresistance was determined as MR(*H*) = (*R*(*H*) − *R*(0))/*R*(0) × 100%, where *R*(*H*) is the electrical resistance in the field *H*, *R*(0) is the electrical resistance in the field *H* = 0. The magnitude of the applied field varied in the range of ±20 kOe.

When studying the Hall effect, the external magnetic field was directed orthogonally to the sample plane (**H***_z_*) and varied within the range of ±20 kOe. The magnitude of the electric current flowing through the microstrip during the measurements was 1 mA. Field dependences of the Hall resistance were obtained in the temperature range of 93–293 K. To minimize the contribution of contact asymmetry (5, 6), the measured Hall voltage values were averaged for the opposite directions of magnetic field and current.

When studying the planar Hall effect, the magnetic field was directed in the film plane. The angle between the magnetic field and direction of electric current (α) was varied in the range of −45–225°. The amplitude of the planar Hall effect was determined as *R*(α) − *R*_0_, where *R*(α) and *R*_0_ are the transverse resistance of the sample in positions α and α = 0°, respectively.

The microstructure of the thin films was investigated using a DRON-3M automated X-ray diffractometer (Russia) with Co Kα radiation. Surface topography was investigated using a Solver Next atomic force microscope (NT-MDT, Russia) operating in semi-contact mode. Measurements were performed under ambient conditions (20 °C, ≈25% relative humidity) using HA-NC silicon cantilevers with a tip radius smaller than 10 nm, a resonance frequency of 235 kHz, and a force constant of 12 N/m. Scans of 1 × 1 µm^2^ areas were acquired at a 1 Hz rate with a 5–10 nm step. The obtained images were analyzed using Nova and Gwyddion 2.55 software.

## Data Availability

All data that supports the findings of this study is available in the published article and/or the supporting information of this article.
